# MINI-DROID-SLAM: Improving Monocular Visual SLAM Using MINI-GRU RNN Network

**DOI:** 10.3390/s25175448

**Published:** 2025-09-03

**Authors:** Ismaiel Albukhari, Ahmed El-Sayed, Mohammad Alshibli

**Affiliations:** 1 Department of Computer Science and Engineering, University of Bridgeport, Bridgeport, CT 06604, USA; ialbukha@my.bridgeport.edu; 2 Department of Electrical and Computer Engineering, University of Bridgeport, Bridgeport, CT 06604, USA; 3 Department of Computer Systems, SUNY Farmingdale State College, Farmingdale, NY 11735, USA; alshibm@farmingdale.edu

**Keywords:** SLAM, Deep Learning, Monocular-SLAM, Bundle Adjustment, Mini-GRU, CONV-GRU, DROID-SLAM, Visual-SLAM

## Abstract

Recently, visual odometry and SLAM (Simultaneous Localization and Mapping) have shown tremendous performance improvements compared to LiDAR and 3D sensor techniques. Unfortunately, attempts to achieve these improvements always face numerous challenges due to their complexity and insufficient compatibility for real-time environments. This paper presents an enhanced deep-learning-based SLAM system, primarily for Monocular Visual SLAM, by utilizing a Mini-GRU (gated recurrent unit). The proposed system, MINI-DROID-SLAM, demonstrates significant improvements and robustness through persistent iteration of the camera position. Similar to the original DROID SLAM, the system calculates pixel-wise depth mapping and enhances it using the BA (Bundle Adjustment) technique. The architecture introduced in this research reduces the time used and computation complexity compared to the original DROID-SLAM network. The introduced model is trained locally on a single GPU using monocular camera images from the TartanAir datasets. The training time and reconstruction metric, assessed using ATE (Absolute Trajectory Error), show robustness and high performance compared to the original DROID-SLAM.

## 1. Introduction

**Figure 1:**
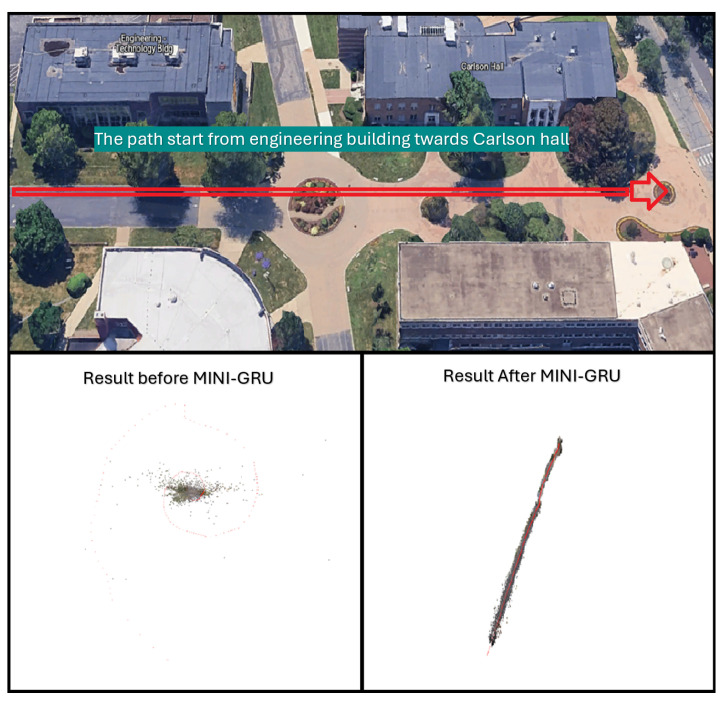
Result of DROID-SLAM (to the **left**), and MINI-DROID-SLAM (to the **right**) on a real-time example.

Simultaneous Localization and Mapping (SLAM) is an indispensable technology that enables autonomous systems to concurrently construct a map of an unknown environment while simultaneously determining their own position within that map. This dual capability is fundamental for navigation and understanding new environments. SLAM systems commonly integrate data from multiple sensor modalities to enhance map quality and robustness. These sensors typically include cameras (monocular and stereo), depth sensors, and Light Detection and Ranging (LiDAR). The incorporation of advanced algorithmic techniques, such as those that support dense reconstruction or feature visualization, significantly improves the system’s ability to recognize and adapt to novel environments. In essence, SLAM empowers autonomous agents to navigate and comprehend their surroundings by concurrently localizing themselves within a newly generated map [1]. A prominent variant of SLAM is Visual SLAM (VSLAM), which heavily relies on visual sensors for its operations. VSLAM systems typically utilize data from monocular cameras, stereo cameras, and, in some cases, LiDAR, to achieve robust performance. A key strength of VSLAM lies in its ability to generate real-time maps by leveraging distinctive visual features extracted from the environment. This characteristic makes VSLAM particularly well-suited for applications demanding high fidelity and real-time environment discovery. Both general SLAM and VSLAM methodologies are critically dependent on their abilities to construct new maps robustly, a vital capability for applications such as autonomous vehicles and drones [2]. This research highlights a clear trend towards more efficient, accurate, and robust SLAM systems powered by deep learning. Figure 1 shows the SLAM output for a real-time sample where DROID-SLAM failed to converge to the correct environment map, while MINI-DROID-SLAM was used to build an accurate map for the new environment. The main contributions of the paper can be summarized as follows:

- Introduction of a new DROID-SLAM architecture based on the Mini-GRU RNN network.
- Improvement on the training performance on the newly proposed model.
- Reduction in the minimum hardware requirements from 4 GPUs to 1 GPU.
- Introduction of MINI-DROID-SLAM, a lightweight DROID-based Monocular-SLAM system that is suitable for real-time requirements.

This proposed work maintains the robustness of deep SLAM frameworks while making them more accessible for real-time systems and embedded applications. For validation, this paper evaluates MINI-DROID-SLAM on various datasets using a monocular camera, as shown in the real-world example in Figure 1, the TUM-RGB dataset, the EuRoC dataset, and finally on the synthetic TartanAir dataset, demonstrating competitive performance and significantly reduced computational overhead.

## 2. Related Works

Recent research has significantly advanced Simultaneous Localization and Mapping (SLAM) systems through the integration of deep learning methodologies, yielding enhanced performance. A notable example is the application of Visual SLAM (VSLAM) techniques for constructing dense 3D maps, as demonstrated with datasets like TartanAir [3].

In 2022, Zachary Teed and Jia Deng introduced DROID-SLAM [4], a pioneering framework that fuses deep learning with traditional SLAM paradigms to achieve highly accurate and robust VSLAM. DROID-SLAM’s architecture is built upon a recurrent iterative update mechanism, inspired by the RAFT model [5], which refines camera poses and pixel-wise depth estimations. The system then employs Dense Bundle Adjustment (DBA), enabling it to process a diverse range of input types, including monocular, RGB-D, and stereo data. To manage inter-frame relationships and dependencies within video streams, DROID-SLAM utilizes a Frame Graph Representation, which facilitates efficient loop closure and optimization. A core innovation lies in its deep learning model, which extracts updates for poses and depth by minimizing geometric errors through state-of-the-art optical flow estimation, to refine predictions iteratively. The model’s efficacy was validated across multiple datasets, including TartanAir, EuRoC, TUM-RGBD, and ETH3D-SLAM, demonstrating robust performance without requiring any retraining. Although it was initially trained on four NVIDIA 3090 GPUs for approximately one week using the TartanAir dataset, the model’s efficacy was demonstrated across various datasets.

In late 2024, the same authors presented DPV-SLAM, an evolution of their DROID-SLAM approach, with a primary focus on improving the efficiency of the loop closure mechanism, while DROID-SLAM incorporated both proximity-based and classical loop closure techniques (image retrieval and pose graph optimization for scale drift correction), DPVO’s redesigned architecture significantly enhances efficiency. This new design enables DPV-SLAM to operate effectively on a single GPU, supporting high frame rates and minimizing memory consumption. Comparative analyses against DROID-SLAM revealed that DPV-SLAM achieves comparable accuracy while being 2.5 times faster, with no observed failures in either indoor or outdoor environments. It is worth noting that DPV-SLAM is an extended version of the DPVO visual odometry system [6].

To continue, several other deep-learning-based SLAM systems have emerged, each with unique contributions, including the following types: iMAP, which utilizes a multi-layer perceptron (MLP) for inferring scene representation and rendering [7]. This system employs RGB-D cameras for real-time training, eliminating the need for prior data to construct dense 3D occupancy and color models. iMAP integrates key frame structures, multi-processing, and dynamic pixel sampling to achieve efficient tracking and global map updates. It excels in scene representation, detail control, and filling unobserved areas, often surpassing traditional SLAM systems in completeness and memory efficiency. Its real-time training capability marks a significant step towards dense real-time SLAM with incremental training and implicit neural representation. Volume-DROID is a real-time SLAM system that combines volumetric mapping with the DROID-SLAM framework [8]. It processes both RGB-D and monocular camera inputs to create 3D maps and accurately track robot localization. A key innovation is the real-time integration of DROID-SLAM with the ConvBKI algorithm, which optimizes data processing efficiency. The system leverages optimized camera positions and point clouds from RGB-D frames to enhance mapping accuracy for autonomous navigation, while evaluations on the TartanAir dataset showed promising performance, challenges with semantic segmentation were observed due to label mismatches. DVI-SLAM enhances accuracy and reliability by integrating visual and inertial (IMU) data, a feature shared with prior deep learning methods, such as DROID-SLAM [9]. This hybrid model uniquely blends various visual data types, adjusting their impact based on confidence levels, resulting in a significant reduction in tracking errors for moving objects within datasets such as TartanAir and EuRoC. DVI-SLAM demonstrates flexibility across different sensor configurations and has been successfully tested on both datasets and real-time data. However, identified limitations include processing speed, memory usage, and sensor integration complexities.

Further enhancements to the DROID-SLAM framework for visual odometry have involved integrating Global Self-Attention and Atrous Spatial Pyramid Pooling (ASPP) into its Conv-GRU model [10]. This modification expands the receptive field, enabling improved optical flow estimation in challenging environments, while the original DROID-SLAM occasionally outperforms these modifications on specific datasets, these enhancements generally improve DROID-SLAM’s accuracy and memory efficiency. GO-SLAM is a deep-learning-based real-time SLAM system designed for reliable 3D reconstruction using RGB-D, monocular, or stereo camera inputs [11]. It reduces trajectory errors through online loop closing and full Bundle Adjustment, achieving superior performance compared to earlier techniques such as DROID-SLAM and iMAP. Operating at 8 FPS with 18 GB of GPU RAM, GO-SLAM demonstrates strong performance in large-scale environments. Optimal performance is achieved through careful key frame selection and loss configurations, leading to cutting-edge results in position estimation and 3D reconstruction across various datasets. Rover-SLAM is a real-time visual-SLAM framework that incorporates deep learning to enhance performance in challenging conditions [12]. It supports diverse camera configurations, including monocular, stereo, monocular–inertial, and stereo–inertial data. The framework utilizes advanced feature extraction and matching algorithms, specifically SuperPoint and LightGlue, to enhance adaptability in dynamic lighting conditions and weakly structured environments. Rover-SLAM achieves high localization accuracy and robust tracking performance comparable to existing SLAM systems. SPAQ-DL-SLAM (Structured Pruning and Quantization) is an optimization framework developed for deep learning SLAM models, particularly DROID-SLAM, to enable their deployment on resource-constrained devices [13]. This optimized version achieves a 20% reduction in model size and an 18.9% decrease in computational complexity, while improving accuracy by 10.5% on the TUM-RGBD dataset. For the SPAQ-DL-SLAM, the enhancements stem from two main steps: structured pruning, which reduces computational demands, and post-training quantization (PTQ), which converts the model’s data from 32-bit to 8-bit integers, maintaining accuracy while improving hardware efficiency; while it is effective across various datasets, this approach struggles in environments with high angular velocity. On the other hand, several newer versions of the GRU modules have been recently introduced, as in [14], where the authors claim a lightweight architecture, yet they still utilize activation functions like Tanh in the input state. Additionally, the proposed models in their research are also used for other applications, rather than SLAM systems [14]. Moreover, in 2025, another research group used a multiscale GRU. For that model, the authors are still using a large number of parameters [15], even more than the original GRU. However, as will be introduced in the next section, the MINI-GRU model is a lightweight RNN network designed for sequential data, such as in Visual SLAM systems. The main concept of MINI-GRU is to reduce the parameters from the original GRU blocks. As a result, MINI-CONV-SLAM, the convolutional version of the MINI-GRU, achieves high performance and accuracy while reducing computational complexity.

## 3. Background

### 3.1. RNN

**Figure 2:**
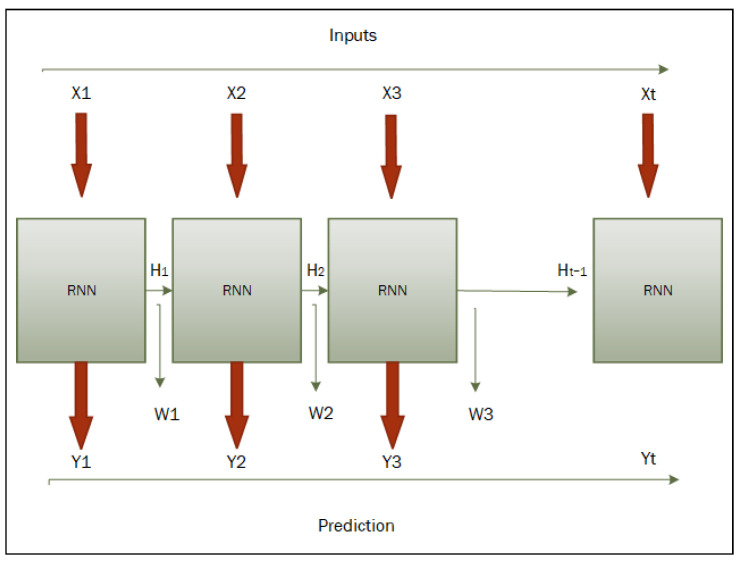
Basic RNN block diagram with the sequence input (X), hidden state (H), output sequence (Y), and weight (W) [16].

Recurrent Neural Networks (RNNs) [16] are a specialized type of neural network (NN) [17] architecture designed to process sequential data by retaining a portion of information from previous inputs. So, this technique is received via many cycles in the network to allow information to be fed back into itself (hidden layers). This feedback mechanism characterizes the RNNs from feed-forward neural networks, which process inputs in a single pass without cycles. Moreover, RNNs are specialized and potent in applications that involve sequence prediction and temporal patterns, such as language interpretation, speech recognition, image descriptions, and video labeling. The architecture of RNNs allows them to consider both the current input and the historical context, which is crucial for understanding sequences where the order of data points is considerable. Figure 2 shows the basic architecture for the vanilla RNN network for input sequence processing.

### 3.2. LSTM

**Figure 3:**
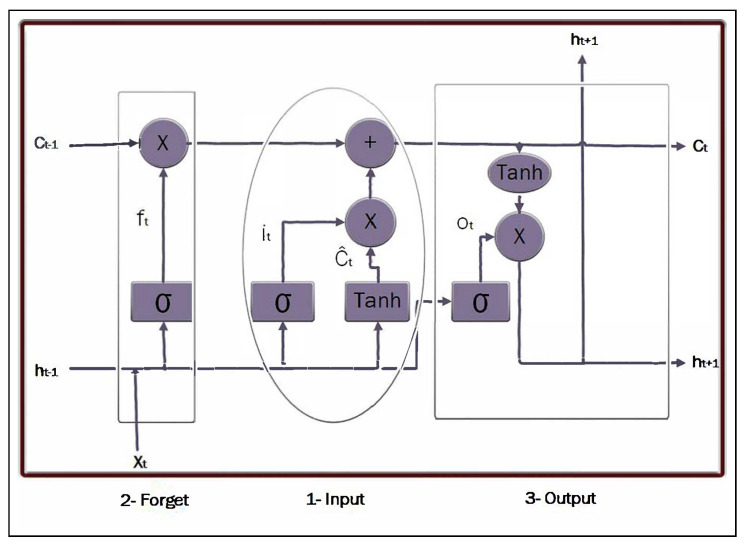
Single LSTM block diagram with the three utilized gates, input gate, forget gate, and output gate [19].

In 1997, Sepp and Schmidhuber presented LSTM (Long Short-Term Memory) [18], a new development of RNN at the time [16]. Their method was designed to overcome the limitations of vanilla RNN, such as the gradient vanishing or explosion problems, by integrating the input and bypassing the gates. These gates were designed to capture long-term dependencies in sequence data, making them particularly effective for tasks that integrate long sequences of text. Figure 3 presents the internal architecture and gates of the LSTM module. In the figure, the arrow connection is a linear fully connected network architecture.

### 3.3. GRU

**Figure 4:**
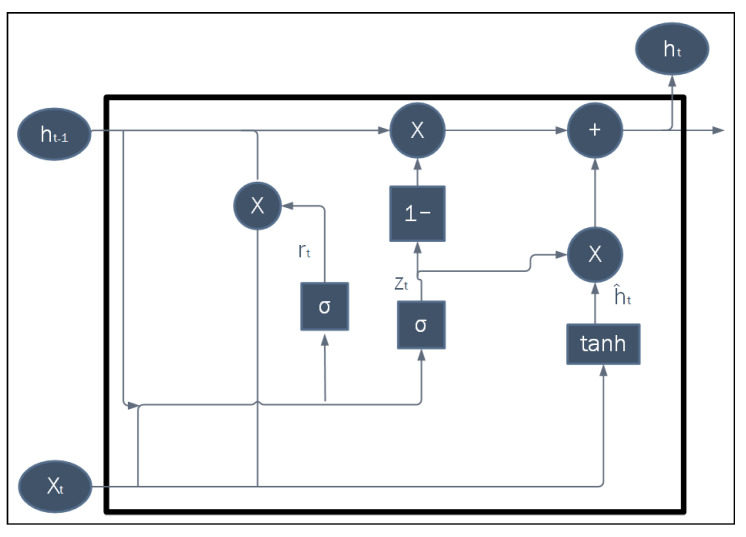
Basic GRU block diagram showing the reset and update gates’ connections.

A gated recurrent unit (GRU) is an advanced version of the RNN architecture, designed to handle data sequences such as speech or text translation. However, it addresses some limitations of the original RNN, such as vanishing gradients and long-term dependency problems. In addition, GRUs provide a gate model to control the flow of information to all networks, to learn what to memorize, what to forget, and what to update. The last main advantage of GRU is its support for long-term dependence, which can be utilized more effectively compared to RNNs and LSTMs [20]. Figure 4 shows the basic architecture and gates of the GRU module. CONV-GRU is an expanded version of the original GRU that incorporates the convolutional process into the module for computer vision applications.

#### Traditional CONV-GRU (Modified from the Original GRU Module)


(1)ht=(1−zt)⊙ht−1+zt⊙h^t(2)zt=σConv2d([xt,ht−1])# Input and hidden state(3)rt=σConv2d([xt,ht−1])# Reset gate (same as previous step)(4)h^t=tanhConv2d([xt,rt⊙ht−1])

### 3.4. MINI-GRU

**Figure 5:**
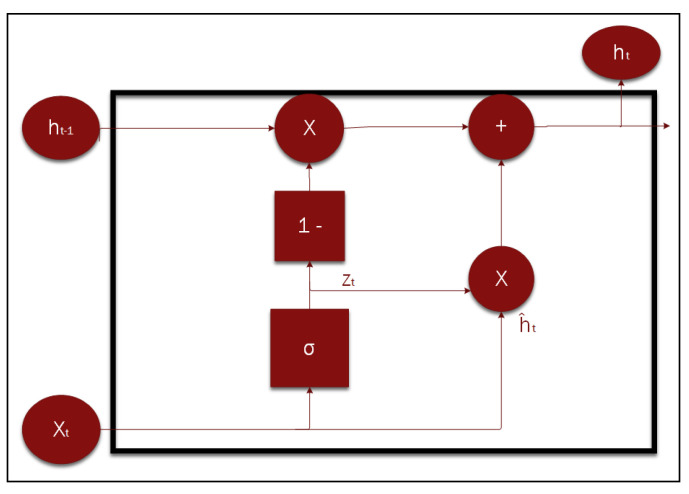
Basic MINI-GRU block diagram that shows the hidden state, input, and output.

Mini-GRU [21] is a light version of the regular GRU, where the reset gate is removed and the other steps are simplified by eliminating the hyperbolic tangent (Tanh) activation function [16]. Moreover, it is also used to process sequences, such as time series data and language translation, among others. Notably, MINI-GRU is lighter and more efficient for real-time applications, featuring fewer gates and parameters, which results in a lower memory footprint. As a result, MINI-GRU achieves much faster training and execution time and is efficient on most datasets. Figure 5 presents the block diagram of the state-of-the-art MINI-GRU RNN module and shows the effective connections after removing the reset gate. A comparison between the original GRU and MINI-GRU modules from different computational and performance perspectives is shown in Table 1. Similar to CONV-GRU, MINI-CONV-GRU is a modified version of the MINI-GRU module that is introduced in this paper for computer vision applications. The following equations explain the details of the proposed module.

#### MINI-CONV-GRU (Modified)

Reset gate removed with the Tanh activation function.


(5)ht=(1−zt)⊙ht−1+zt⊙h^t(6)zt=σConv2d([xt,ht−1])# Input and hidden state(7)h^t=Conv2d(xt)

## 4. Methodology

**Figure 6:**
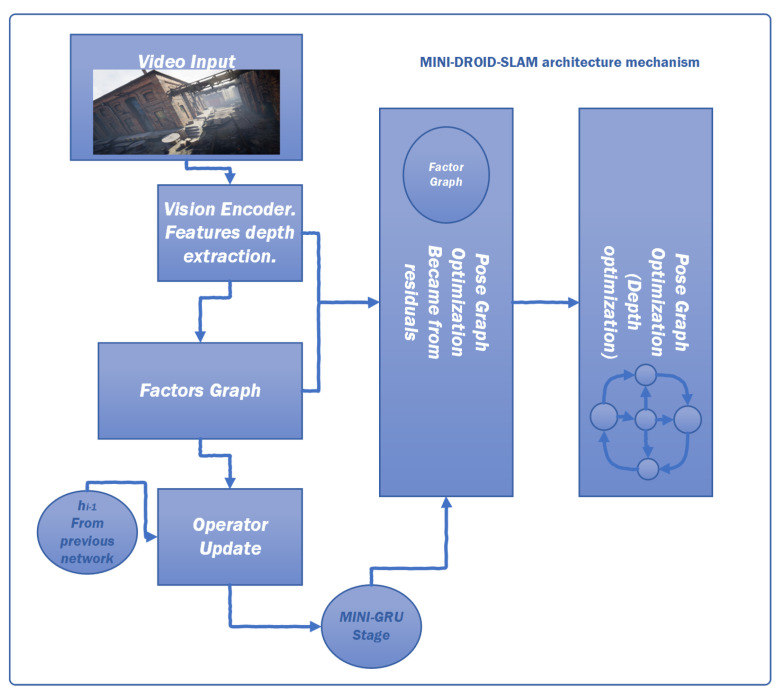
MINI-DROID-SLAM system with the usage of MINI-CONV-GRU models.

**Figure 7:**
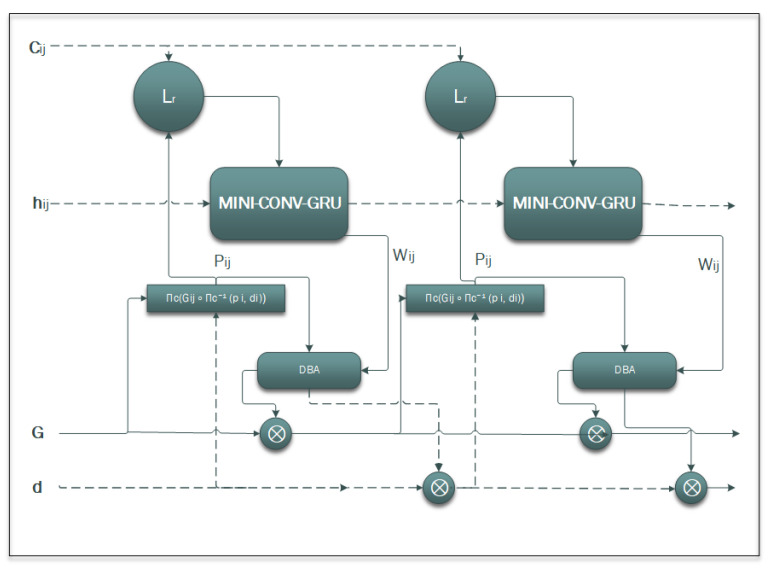
Operation update. Where Lr is correlation lookup, MINI-CONV-GRU has no reset gate (r), Pij is projection, and DBA is Dense Bundle Adjustment.

**Figure 8:**
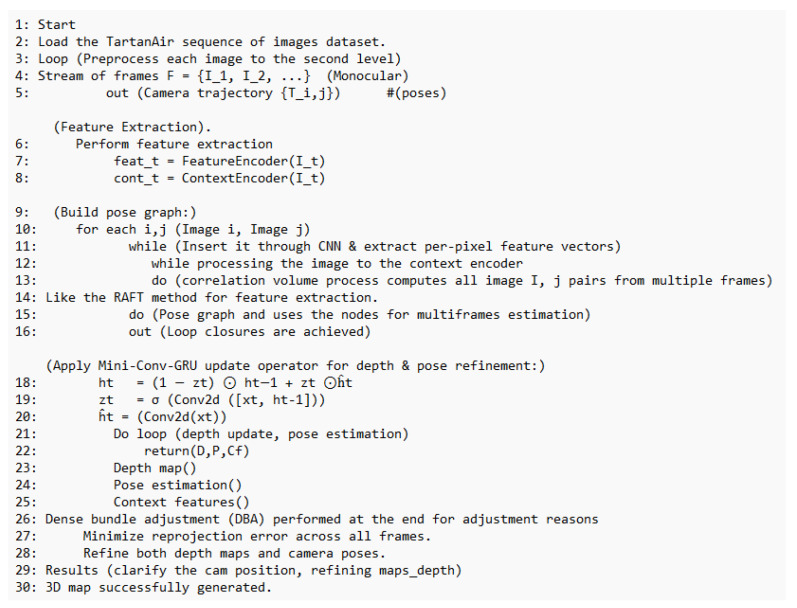
MINI-DROID-SLAM system workflow.

Considering the advantages of the MIN-GRU presented in the previous section, this paper proposes enhancements and modifications to the DROID-SLAM deep learning Simultaneous Localization and Mapping (SLAM) system. Specifically, it replaces the utilized CONV-GRU module with a modified version of the state-of-the-art MINI-GRU module, known as MINI-CONV-GRU. Table 2 presents a comparison between fully connected neural networks (Basic NN), CONV-GRU, and MINI-CONV-GRU modules, focusing on their suitability for real-time applications. The proposed system utilizes the BA (Bundle Adjustment) for pixel-wise depth, precisely as the DROID-SLAM technique does. The proposed system architecture is shown in Figure 6. The proposed system targets monocular camera data; therefore, only monocular Tartanair datasets will be used in the training process. The process starts by extracting features from the input image. The features are extracted using a network of six residual blocks and three downsampling layers similar to the original DROID-SLAM architecture to produce a dense feature map that is used to build the $\left(C_{ij}\right)$, input to the update process, as shown in Figure 7. Those correlation features are indexed using the $\left(L_{r}\right)$, correlation lookup operator. Another context network works on the input image to produce context features that are also used in the output update step. Similar to the original technique, the dense corresponding field $\left(P_{ij}\right)$ is calculated and used in both the indexing process and the BA step. Figure 8 illustrates the process in algorithmic steps.

### Update Operator in MINI-DROID-SLAM

After the input sequences are processed for feature extraction and indexing, the data are applied to the MINI-CONV-GRU to produce updated information for the output level. Figure 7 illustrates the *update operator*, which is the central core of MINI-DROID-SLAM, utilizing the MINI-CONV-GRU module, and is responsible for refining camera poses and dense depth maps iteratively. Unlike the original MINI-GRU, the MINI-CONV-GRU concatenates and uses the input of the previous layer $\left(h_{ij}\right)$ to calculate the $\left(z_{ij}\right)$ gate. The update operator acts on edges of the frame graph, where each edge connects two frames (i) and (j) that have overlapping views.

As explained earlier, correlation volumes are computed from dense feature maps of the two frames, representing similarity scores between all pairs of pixels. Context features extracted from the images provide additional information to guide updates. The hidden state $\left(h_{ij}\right)$ of the recurrent unit carries the previous memory across iterations. The update operator is implemented mainly as a 3×3 MINI-CONV-GRU. At each iteration (k), it updates its hidden state based on the inputs and outputs flow revisions, which are corrections to the current optical flow estimates. The process inside the MINI-CONV-GRU can be stated as follows:

- **Step 1:** Concatenate the new input indexed correlation features $\left(C_{ij\_ind}\right)$ sequence along the second dimension (dim = 1).
- **Step 2:** Concatenate the $\left(h_{ij}\right)$ and the indexed $\left(C_{ij\_ind}\right)$ tensors along the second dimension to form $\left(h_{ij\_ind}\right)$.
- **Step 3:** Obtain the shape of the $\left(h_{ij}\right)$ tensor, denoted as (b,c,h,w).
- **Step 4:** Apply a sigmoid activation function to the output of a weighted layer *w* applied to $\left(h_{ij}\right)$, then perform element-wise multiplication with $\left(h_{ij}\right)$, which results an intermediate variable $\left(glo_{ij}\right)$.
- **Step 5:** Reshape the resulting tensor $\left(glo_{ij}\right)$ to a 3D tensor, compute the mean along the last dimension, and reshape it back to a 4D tensor.
- **Step 6:** Apply a sigmoid activation function to the output of a convolutional layer applied to $\left(h_{ij\_ind}\right)$, the output of this step is the $\left(z_{ij}\right)$.
- **Step 7:** Compute an intermediate step variable $\left(q_{ij}\right)$ by summing the outputs of two parallel convolutional layers:
  – convq, by appling 3×3 convolutional filters to the concatenated $\left(h_{ij\_ind}\right)$ tensor.
  – convq_glo, by applying 1×1 convolutional process for channel adjustments to the $\left(glo_{ij}\right)$ tensor.
- **Step 8:** Update the $\left(h_{ij}\right)$ tensor using the calculated $\left(z_{ij}\right)$ and $\left(q_{ij}\right)$ as follows:
  $$h_{ij}=\left(1-z_{ij}\right)\times h_{ij}+z_{ij}\times q_{ij}$$
- **Step 9:** Return the updated $\left(h_{ij}\right)$ variable tensor, and repeat **Step 2** again with the new input indexed correlation.

The predicted flow revisions outputs from the update operator, along with the dense corresponding field $\left(P_{ij}\right)$, are passed to the Dense Bundle Adjustment (DBA) layer. The DBA performs a differentiable Gauss–Newton optimization that jointly updates camera poses and dense depth maps. This process tightly couples pose and depth refinement, enforcing geometric consistency across frames. At each iteration, the current pose and depth estimates are used to compute dense correspondences between frames, which inform the next update. Lastly, the operator works on the edges of a frame graph that encodes co-visibility between frames.

## 5. Results

**Figure 9:**
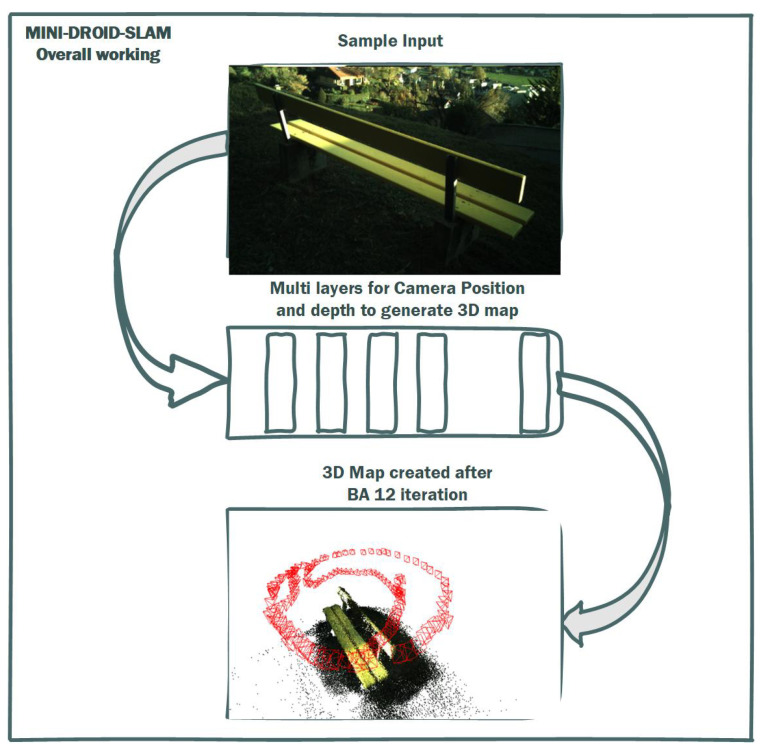
Building a dense 3D map of the unknown environment and simultaneously localizing using estimated camera positions. The generated trajectory is presented in red.

As stated earlier, the proposed MINI-DROID-SLAM system is trained on monocular images from the TartanAiR dataset, with a batch size of 1250 steps, a resolution of 384 × 512, 7-frame clips, and 12 iterations for BA, rather than the original DROID-SLAM. The machine used for this work has the following specifications: an Intel Core i9 processor, 32 GB of RAM, and a single GPU, the RTX 3090 with 24 GB of memory. The structure of applying input data sequence to the proposed architecture is shown in Figure 9. Table 3 shows the testing results of DROID-SLAM against the proposed MINI-DROID-SLAM model on the monocular benchmark TarTanAir dataset. The data utilized for the benchmark are not the same as those used for training.

**Figure 10:**
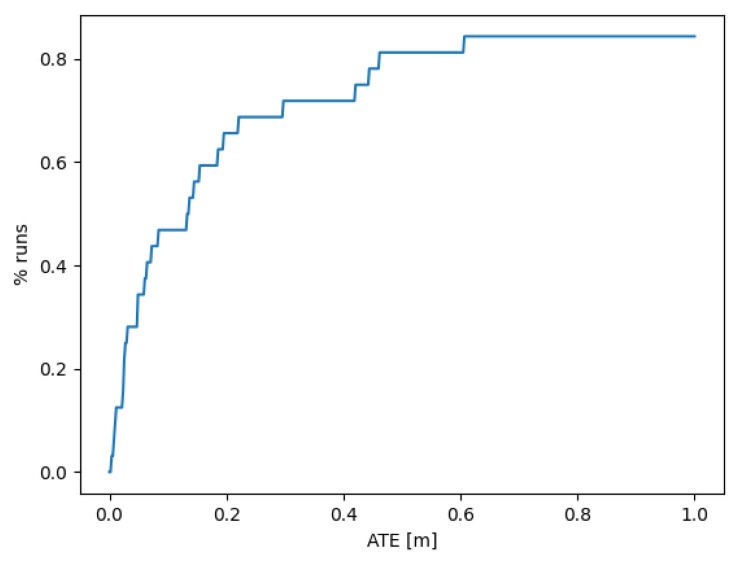
The number of successful trajectories as a function of the Absolute Trajectory Error (ATE) for the output of the trained model on the validation group of the TartanAir dataset.

Figure 10 presents the ablation experiment on TartanAir validation split dataset to show the advantage of the proposed MINI-DROID-SLAM over the original DROID-SLAM.

**Figure 11:**
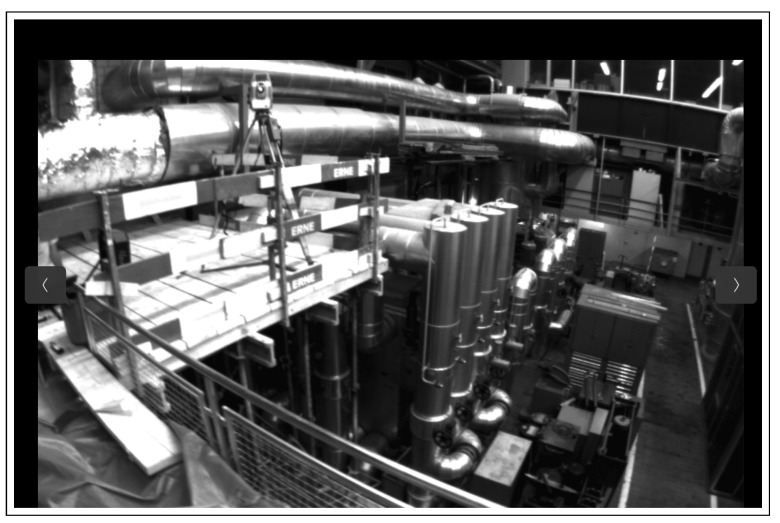
Sample scene from the MAV0 sequence of the EUROC dataset.

**Figure 12:**
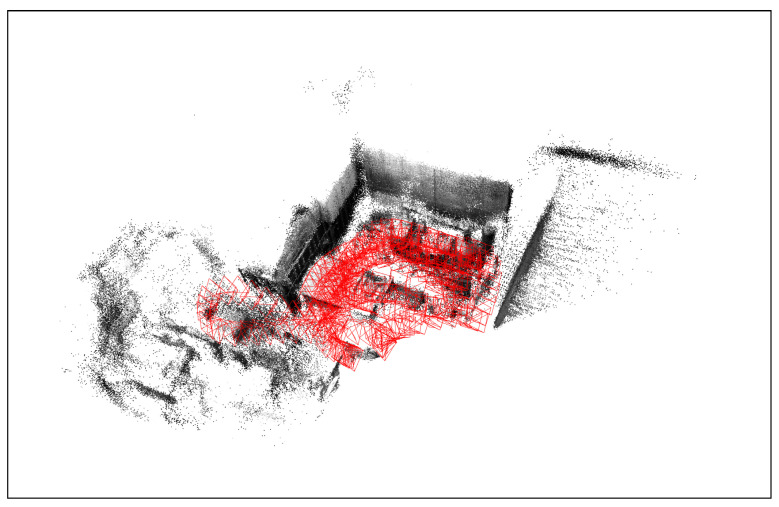
Generated 3D map and trajectory for the EuRoC MAV0 sequence using MINI-DROID-SLAM model. The generated trajectory is presented in red.

For further verification, the proposed model is tested on the same dataset used for testing in the original DROID-SLAM paper, specifically the EuRoC and the TUM datasets. A sample from the EuRoC dataset with our output SLAM result is shown in Figure 11 and Figure 12, respectively.

The complete comparison between generated maps and localization between DROID-SLAM, ORB-SLAM3, and MINI-DROID-SLAM on EuRoC and TUM monocular RGB datasets is shown in Table 4 and Table 5. These results demonstrate that MINI-DROID-SLAM generates maps and trajectories for the EuRoC and TUM datasets with the same accuracy as DROID-SLAM, but with improved execution time.

**Figure 13:**
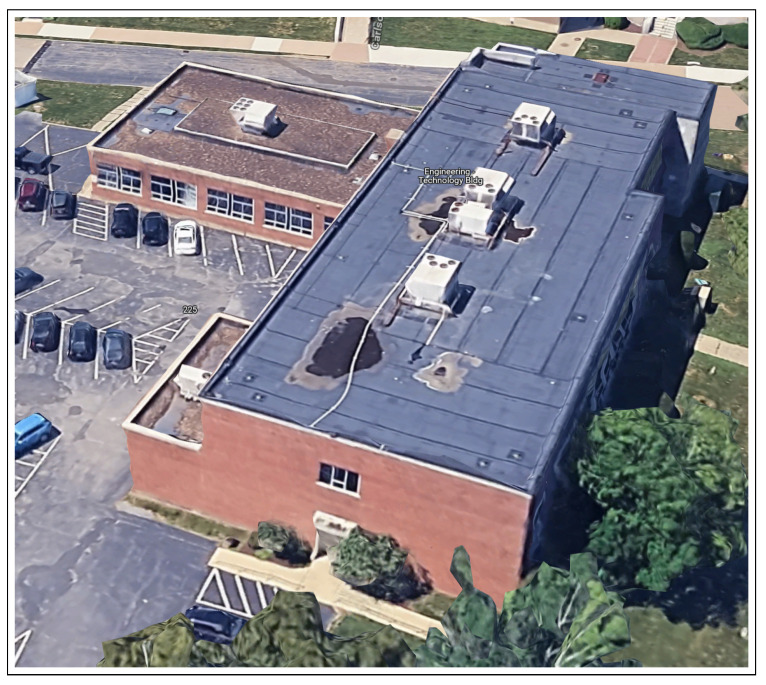
Engineering and Technology Building of the University of Bridgeport map from Google Maps.

**Figure 14:**
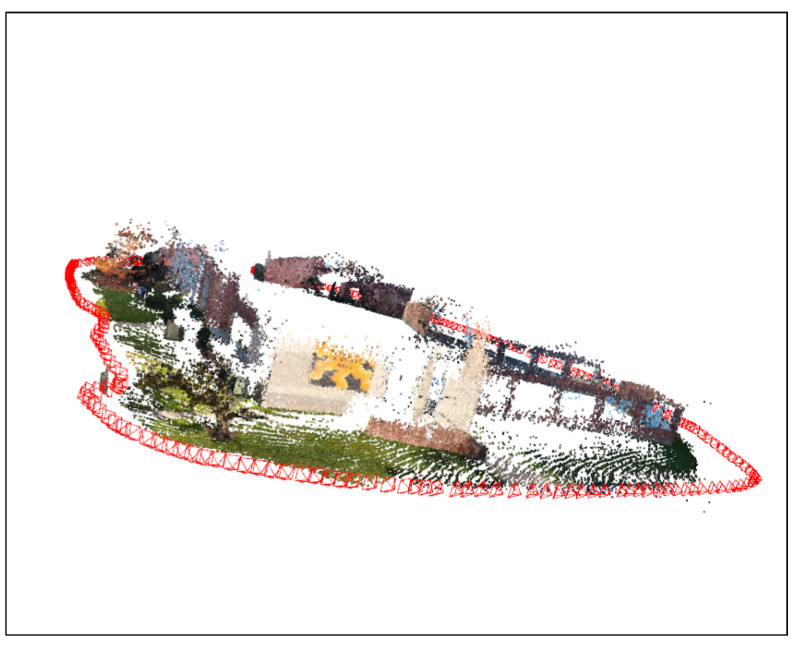
Three-dimensional construction of the Engineering and Technology Building using MINI-DROID-SLAM trained model. The generated trajectory is presented in red.

Moreover, we also applied the MINI-DROID-SLAM model to real-time data collected from the local campus of the University of Bridgeport to test its real-time capabilities, map building, and trajectory generation. The generated map and trajectory matched the results collected from the original DROID-SLAM technique, but better frame rates per second were achieved on one GPU compared to the original DROID-SLAM. Figure 13 and Figure 14 show the map built for the campus building. As shown earlier in Figure 1, in some cases, MINI-DROID-SLAM shows more robust and better performance compared to the original DROID-SLAM.

**Figure 15:**
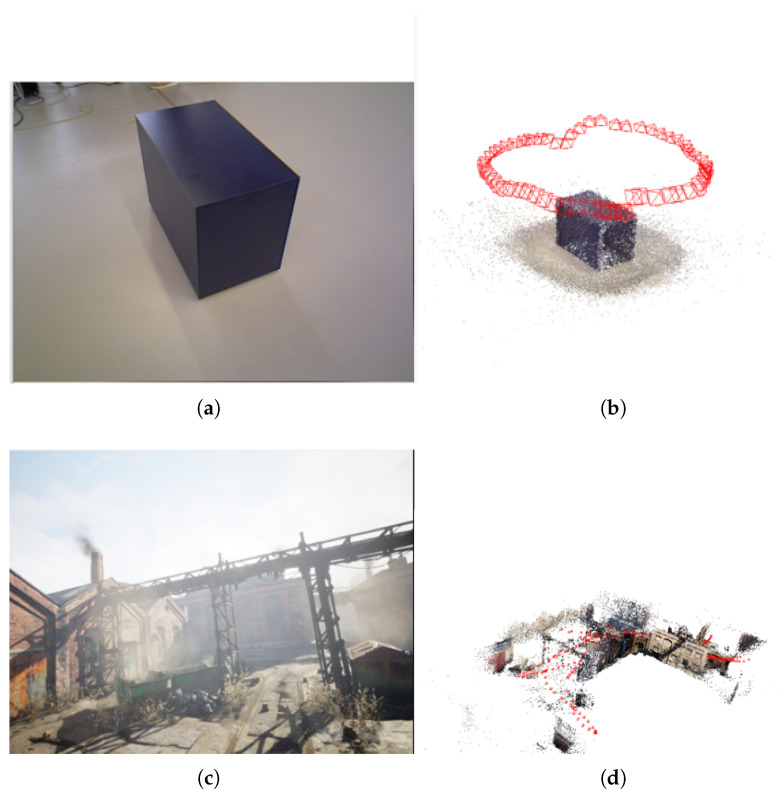
Examples of collected results from several datasets utilized for SLAM tasks. For these samples, a single GPU 3070 RTX running 12 BA iterations has been used: (**a**) Cabinet TUM-RGB stream dataset [25]. (**b**) Results show the map and trajectory collected from the trained model for the Cabinet example. The generated trajectory is presented in red. (**c**) Abandoned Factory scenario from TartanAir [3] dataset. (**d**) The result of map building and localization for the Abandoned Factory scenario. The generated trajectory is presented in red.

Finally, to demonstrate the scalability of the trained model, the TartanAir validation dataset is tested on a single lower-end GPU, specifically an RTX 3070 with 8 GB of memory. The results show that the proposed model runs on the GPU without encountering an out-of-memory error, which is a common issue with the original DROID-SLAM model. Figure 15 shows the collected results from the RTX 3070 GPU on samples from both TartanAiR and TUM datasets. For this demonstration, the input sequence has been processed at a rate of 7.75 iterations per second, with a memory utilization of 6.2 GB.

## 6. Discussion

The MINI-DROID-SLAM system represents a significant advancement over conventional SLAM techniques, particularly in comparison to its predecessor, DROID-SLAM. This progress is attributed to the integration of a MINI-CONV-GRU module within the feature map and camera pose update processes. This architectural optimization yields substantial improvements in both computational efficiency and mapping accuracy. Evaluations conducted on the TartanAir synthetic dataset and real-world “campus building” data demonstrate MINI-DROID-SLAM’s capability to construct highly accurate 3D maps with reduced trajectory error, and improved real-time performance. The system exhibits performance comparable to established SLAM frameworks, such as ORB-SLAM and DROID-SLAM, particularly in RMSE-based ATE evaluations across various sequences. The robustness and sustained performance of MINI-DROID-SLAM are maintained through the application of BA and a lightweight recurrent module. This design facilitates efficient training and real-time inference on a single high-end RTX GPU. The successful implementation of MINI-DROID-SLAM underscores the potential for architectural optimizations, such as the MINI-GRU, to achieve efficient and accurate SLAM solutions, even with limited hardware resources, thereby rivaling state-of-the-art frameworks trained with significantly greater computational power. This evidence supports the hypothesis that innovative architectural designs can lead to robust SLAM solutions for real-world applications. Table 6 presents a comprehensive comparison of DROID-SLAM, DPV-SLAM, and the proposed MINI-DROID-SLAM algorithm. This analysis highlights the advancements of the proposed technique over existing state-of-the-art algorithms, as evidenced by various performance metrics.

**Figure 16:**
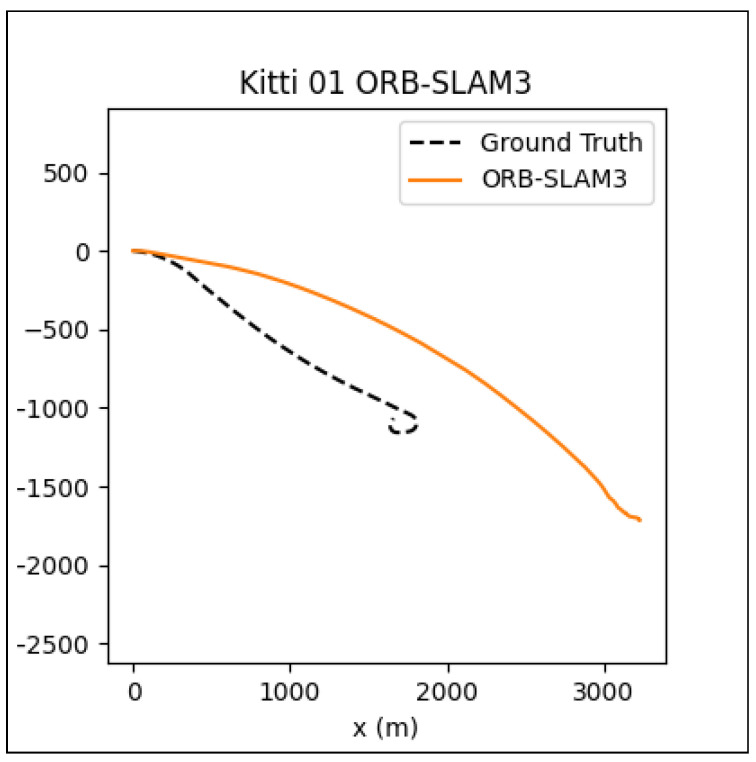
ORB-SLAM3 ATE on Kitti 01 sequence = 661.87.

**Figure 17:**
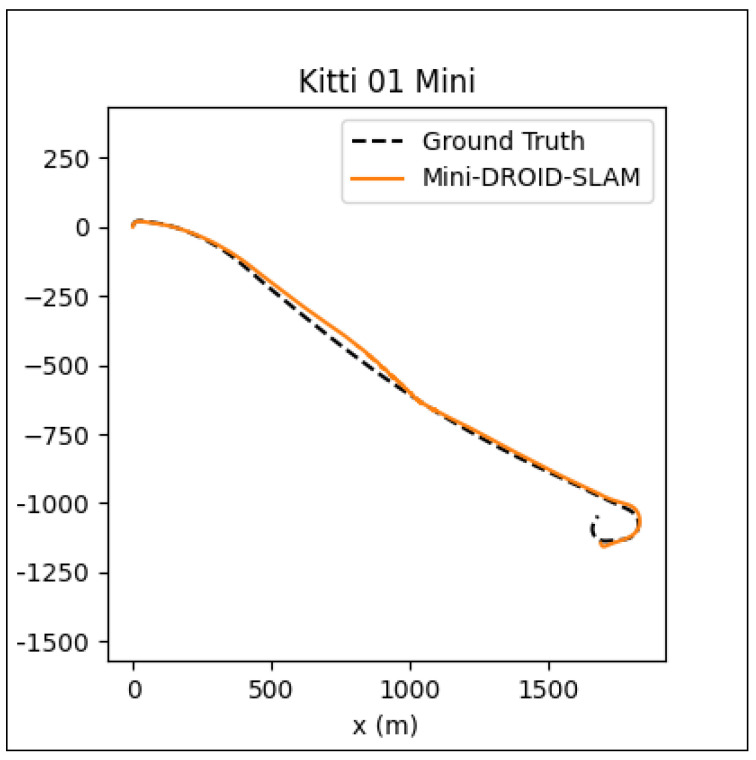
MINI-DROID-SLAM ATE on Kitti 01 sequence = 56.24.

Although the MINI-DROID-SLAM shows improved results over DROID-SLAM, the proposed model still requires execution on a GPU to enhance quality and performance in both training and testing times. On the other hand, compared to the original DROID-SLAM, the proposed technique requires a lower GPU footprint and can be executed on a lower-latency GPU, such as the RTX 3070. For outdoor environments, although the mapping process can sometimes be a significant challenge and requires further improvement, the camera localization task still performs comparably. To examine the performance of the MINI-DROID-SLAM in outdoor environments, it has been tested against ORB-SLAM3 on one of the common Kitti’s dataset sequences (01). As shown in Figure 16, the ORB-SLAM3 [26] failed to detect the correct camera trajectory due to lighting and challenging environmental conditions. On the other hand, MINI-DROID-SLAM successfully generates the camera trajectory with comparable performance, as shown in Figure 17. Moreover, as stated earlier, Figure 1 shows a comparison between the original DROID-SLAM and MINI-DROID-SLAM, where the original version failed to build a map or find the camera trajectory, compared to the MINI version that worked better regardless of the challenging light conditions (the sun was facing the camera). On the other hand, since MINI-DROID-SLAM inherits the same BA technique from the old DROID-SLAM, it will suffer from the same drawbacks, such as the loop-closure problem. This problem necessitates a reevaluation of the BA system to enhance its quality and further improve its performance.

## 7. Conclusions

This paper has presented MINI-DROID-SLAM, an enhanced VSLAM system that refines the deep learning architecture of DROID-SLAM by integrating a compact and efficient MINI-GRU module. Results indicate that the proposed methodology significantly improves training speed and reduces computational complexity, all while maintaining or surpassing the accuracy of the original DROID-SLAM. The system demonstrates robust performance across diverse environments using monocular input, thereby confirming the viability of lightweight models for real-time SLAM applications. Overall, MINI-DROID-SLAM offers a more accessible and efficient solution for Simultaneous Localization and Mapping, particularly for real-world deployments. Future research can extend the current MINI-DROID-SLAM methodology in several key areas. Expanding its support to include multiple sensor modalities and sensor fusion, such as RGB-D and stereo vision, to enhance its versatility. Furthermore, increasing the diversity of training data to encompass a broader range of real-world environments could significantly improve the model’s generalization capabilities. Moreover, optimizing the model for lower-latency devices and embedded systems by reducing GPU memory consumption is also a crucial direction. Additionally, future work could involve implementing an adaptive learning mechanism for dynamic environments and rigorously evaluating performance under uncertainty and low-light conditions, which would be essential for broader deployment in autonomous vehicle systems. Moreover, explainable AI techniques can be applied to expand the intended flow of the proposed modules.

## Figures and Tables

**Table 1:** Comparison between GRU and MINI-GRU.

Feature	GRU [22]	MINI-GRU
Gates in single block	Update gate ($h_{t-1}$) and reset gate (*r*)	Only update gate, reset gate removed
Parameters and gates	Has many parameters	Fewer parameters
Processing	Slow	Fast
Memory usage	Uses much memory during training	Uses less memory during training
Cases usage	Deep learning (DL) methods, DL-SLAM in regular methods	Deep learning (DL) methods, DL-SLAM with low weights methods

**Table 2:** Comparison of different deep learning techniques used in VSLAM.

Method	Pros	Cons	Realtime
CONV-GRU	Excellent accuracy, less speed	high Complexity	Better time complexity than LSTM
MINI-CONV-GRU	Excellent accuracy, high speed	Less complexity	Better on real-time applications
Basic NN	Fastest with fine accuracy	Lower accuracy	Best for real-time applications

**Table 3:** Comparison of trajectory errors (ATE in meters) across sequences for different scenarios in monocular SLAM methods. Results are collected from the TartanAir monocular benchmark dataset.

Method	MH000	MH001	MH002	MH003	MH004	MH005	MH006	MH007
**(Classic)**								
ORB-SLAM [23]	1.30	0.04	2.37	2.45	–	–	21.47	2.73
**(DL-Method)**								
DROID-SLAM3 [4]	0.08	0.05	0.04	0.02	0.01	1.31	0.30	0.07
MINI-DROID-SLAM	0.08	0.00	0.00	0.00	0.00	0.00	–	–

**Table 4:** Absolute Trajectory Error (ATE) comparison on the EuRoC dataset (lower is better).

Method	MH01	MH02	MH03	MH04	MH05	V101	V102	V103	V201	V202	V203
**(Classic)**											
DSO [1]	0.046	0.046	0.172	3.810	0.110	0.089	0.107	0.903	0.044	0.132	1.152
SVO [24]	0.100	0.120	0.410	0.430	0.300	0.070	0.210	X	0.110	0.110	1.080
DSM [25]	0.036	0.055	0.057	0.067	0.067	0.095	0.059	0.076	0.056	0.057	0.784
ORB-SLAM3 [26]	0.016	0.027	0.028	0.138	0.072	0.033	0.015	0.033	0.023	0.029	X
**(DL-Method)**											
DROID-SLAM [4]	**0.013 **	**0.014**	**0.022**	**0.043**	**0.043**	**0.037**	**0.012**	**0.020**	**0.017**	**0.013**	**0.014**
MINI-DROID-SLAM	**0.013**	**0.014**	**0.022**	**0.043**	**0.043**	**0.037**	**0.012**	**0.020**	**0.017**	**0.013**	**0.014**

**Table 5:** Comparison of different SLAM techniques applied to the monocular TUM-RGB dataset. The numbers represent ATE (m) for various sequences (lower is better).

Method	360	Desk	Desk2	Floor	Plant	Room	Rpy	Teddy	Xyz
**(Classic)**									
ORB-SLAM3 [26]	X	**0.017 **	0.210	X	0.034	X	X	X	**0.009**
**(DL-Method)**									
DROID-SLAM [4]	**0.111**	**0.018**	**0.042**	**0.021**	**0.016**	**0.049**	**0.026**	**0.048**	**0.012**
MINI-DROID-SLAM	**0.111**	**0.018**	**0.042**	**0.021**	**0.016**	**0.049**	**0.026**	**0.048**	**0.012**

**Table 6:** Comparison of DROID-SLAM, DPV-SLAM, and MINI-DROID-SLAM (ours).

Feature	DROID-SLAM [4]	DPV-SLAM [6]	MINI-DROID-SLAM (Ours)
**Method type**	Full SLAM	Visual odometry (VO)	Full SLAM
**Computational cost**	High GPU usage	Lower GPU usage	Lower GPU usage
**Accuracy**	High accuracy	High in VO	High and efficient
**Scalability**	Not real-time application	Suitable for real-time	Better in real-time
**Primary goal**	Full SLAM achieved	Efficient VO	Full SLAM achieved
**Machine setup**	4× GPU 3090	1× GPU 3090	1× GPU 3090
**Training time**	∼7 days	Depends (avg 1–3 days)	∼5 days

## Data Availability

The original data presented in this study are included in the article. Further inquiries can be directed to the corresponding author.

## Abbreviations

MINI-DROID	Minimal-Differentiable Recurrent Optimization-Inspired Design
DL	deep learning
NN	Neural Networks
BA	Bundle Adjustment
SLAM	Simultaneous Localization and Mapping
ATE	Absolute Trajectory Error
